# Recombination landscape shaped by inversion polymorphisms: a high-density linkage map and chromosome-level assembly of inversion-rich spruce bark beetle genome

**DOI:** 10.1093/g3journal/jkag017

**Published:** 2026-01-20

**Authors:** Krystyna Nadachowska-Brzyska, Anna Maryańska-Nadachowska, Dineshkumar Kandasamy, Martin N Andersson, Zuzanna Nowak, Piotr Zieliński, Matias Rodriguez, Wiesław Babik

**Affiliations:** Institute of Environmental Sciences, Faculty of Biology, Jagiellonian University, Gronostajowa 7, Kraków 30-387, Poland; Institute of Systematics and Evolution of Animals, Polish Academy of Sciences, Sławkowska 17, Krakow 31-016, Poland; Department of Biology, Lund University, Kontaktvägen 10, Lund 223 62, Sweden; Max Planck Center Next Generation Insect Chemical Ecology (nGICE), Lund SE-223 62, Sweden; Department of Biology, Lund University, Kontaktvägen 10, Lund 223 62, Sweden; Max Planck Center Next Generation Insect Chemical Ecology (nGICE), Lund SE-223 62, Sweden; Institute of Environmental Sciences, Faculty of Biology, Jagiellonian University, Gronostajowa 7, Kraków 30-387, Poland; Institute of Environmental Sciences, Faculty of Biology, Jagiellonian University, Gronostajowa 7, Kraków 30-387, Poland; Institute of Environmental Sciences, Faculty of Biology, Jagiellonian University, Gronostajowa 7, Kraków 30-387, Poland; Institute of Environmental Sciences, Faculty of Biology, Jagiellonian University, Gronostajowa 7, Kraków 30-387, Poland

**Keywords:** linkage map, recombination rate, inversion-rich genomes, spruce bark beetle

## Abstract

Understanding the recombination landscape is crucial for revealing the extent of its variation across the tree of life and for uncovering its underlying causes and evolutionary consequences. Among the many factors influencing recombination rates, polymorphic inversions are particularly important modifiers. Increasingly, complex inversion landscapes are being documented across diverse taxa, and detailed recombination rate data are essential for advancing our understanding of variation in inversion-rich genomes. Here, we combined whole-genome sequencing of 2 two-generation families with cytogenetic karyotyping to reconstruct a linkage map of the inversion-rich spruce bark beetle (*Ips typographus*) genome. Our results revealed a different chromosome number than previously reported (15AA + Xy) and a recombination landscape strongly shaped by the inversion landscape. The total length of the autosomal, sex-averaged map was 978 cM, with an overall mean recombination rate of 4.9 cM/Mb. Recombination was spatially heterogeneous across the genome and was significantly reduced in parents heterozygous for specific inversion arrangements. We also used the linkage map to upgrade the existing genome assembly to chromosome level, correcting previous misassemblies (often associated with inversions), revising inversion size estimates, identifying new putative inversions, and updating repeat content. Notably, inversions were found to be depleted in transposable elements. These findings provide a valuable foundation for future research on this important forest pest and offer broader insights into how recombination landscapes are shaped in inversion-rich genomes.

## Introduction

Meiotic recombination represents a fundamental biological process in sexually reproducing species. It involves the exchange of genetic material between homologous chromosomes and thus plays a pivotal role in the generation of new allele combinations, thereby influencing patterns of genetic variation and potentially affecting the expression and evolution of traits (Hansen 2006; Cooper 2007; Ortiz-Barrientos et al. 2016; Peñalba and Wolf 2020). The recombination rate can be influenced by a number of factors, including the number of chromosomes and the presence of structural variants or specific genetic loci (e.g. PRDM9 in mammals, Paigen and Petkov 2018). Furthermore, recombination rate can vary across the genome, between sexes, populations, or even individuals of the same species (Ritz et al. 2017; Stapley et al. 2017a; Johnston 2024). Characterization of the recombination landscape is essential for understanding the extent of its variation across the tree of life, its causes and consequences, as well as providing the key parameter used in a wide range of population and evolutionary genetic analyses (Peñalba and Wolf 2020). However, despite its importance, and recent advances in sequencing technologies and analysis methods, recombination rate information is still not available for most species. Moreover, the pace at which new genomes are being sequenced does not match the pace at which recombination rates are being estimated, highlighting the need to provide this information for a variety of taxa.

Inversions, chromosomal mutations that reverse the order of genes within a chromosome, are often referred to as recombination modifiers. This is because polymorphic inversions, present in 2 orientations (ancestral and derived-inverted) within species, significantly suppress recombination in heterozygous individuals (Sturtevant and Beadle 1936). By keeping co-adapted alleles of different genes together, such a mechanism may, for example, facilitate local adaptation in the face of gene flow between populations (Kirkpatrick and Barton 2006; Faria, Johannesson, et al. 2019b; Berdan et al. 2023). Inversions can also affect recombination rates outside their boundaries by extending recombination suppression far beyond inversion breakpoints (Adrion et al. 2020; Koury 2023; Li et al. 2023) or by increasing local recombination rates in regions distant from inversions (Crown et al. 2018). As studies increasingly reveal the complexity of inversion polymorphism landscapes (Wellenreuther et al. 2019; Faria et al. 2019a; Todesco et al. 2020; Harringmeyer and Hoekstra 2022), detailed information on recombination rates is essential for advancing our understanding of recombination variation in inversion-rich genomes.

The Eurasian spruce bark beetle (*Ips typographus* L.) is an emerging model species for forest pests. The species is widely known for its outbreak potential (Seidl et al. 2016; Marini et al. 2017; Biedermann et al. 2019). Its impact on forest ecosystems can be devastating, causing mass mortality of spruce stands in European forests (Liebhold et al. 2017; Bentz et al. 2019; Hlásny et al. 2019). Decades of research on its biology and ecology have recently been complemented by new genomic resources, which have revealed multiple polymorphic inversions in the species' genome (Mykhailenko et al. 2024). Further in-depth analysis of the evolutionary forces maintaining inversion polymorphism and their influence on the genome wide variation patterns is limited by the lack of information on recombination rate variation, as well as the lack of chromosome-level genome assembly (current genome assembly is composed of 272 contigs; N50 = 6.65 Mb; Powell et al. 2021).

Here, we used whole-genome sequencing in 2 families to reconstruct a linkage map of the inversion-rich spruce bark beetle genome. The results suggested a different number of chromosomes than previously reported (Virkki 1960). We therefore used cytogenetic karyotyping to determine the correct number of chromosomes. We estimated the recombination rate across the genome and analyzed it in the context of the complex inversion polymorphism landscape recently identified in the spruce bark beetle. We also used linkage map information to update the existing genome assembly to chromosome level, correcting previous misassemblies, revising inversion sizes and evidence for previously suggested within-inversion double crossover events. The results not only serve as a valuable resource for future studies of this important forest pest but also provide insights on how recombination landscape is shaped in inversion-rich genomes.

## Methods

### Samples

Bark beetles used in this study were cultured in the laboratory using the method described in Anderbrant et al. (1985) with minor adjustments. The starting beetle population was obtained from an infested tree in February 2021 near Asa, Sweden. Briefly, a single male and female from the F5 generation of stock population (maintained at large population size; >1,000 individuals) were allowed to infest freshly cut spruce logs, each placed individually in rearing containers in August 2021. The environmental chamber was set at 23 °C with a relative humidity of 65% and a photoperiod of 20 h per day. After 15 d, larvae and pupae from a single family were manually removed by exposing the bark surrounding the beetle gallery and stored in 95% ethanol.

DNA from 2 beetle families (with 65 and 82 offspring) was extracted using the Wizard Genomic DNA Purification Kit (Promega). DNA from parents and 69 offspring were subject to PCR-free genomic library construction (Illumina TruSeq DNA PCR-free) and DNA from remaining offspring were prepared using Illumina TruSeq DNA Nano protocols. Such a division was motivated by the usage of PCR-free libraries in a separate project aiming at mutation rate estimation (Mykhailenko et al. in preparation). All libraries were subject to 2 × 150 bp paired-end Illumina sequencing (aiming at coverage of 90× and 20× for PCR-free and Nano libraries, respectively). The quality of raw read data was assessed with FastQC (https://github.com/s-andrews/FastQC). Trimmomatic (Bolger et al. 2014) was used to remove low-quality reads and adaptors before mapping reads to the spruce bark beetle genome (Powell et al. 2021) with Bowtie2 (Langmead and Salzberg 2012). Mapping was followed by duplicate reads removal with PicardMarkDuplicates (https://broadinstitute.github.io/picard/). To equalize coverage among samples we downsampled bam files from highly covered individuals using Picard DownsampleSam (https://broadinstitute.github.io/picard/). Repeats were masked using bedtools (intersect, Quinlan 2014), and variant calling was performed using samtools mpileup with minimum mapping quality (-q) 10 and minimum base quality (-Q) 10 (Li 2009) following Lep-Map3 protocol (Rastas 2017); see below).

### Inversion genotyping in parents

To be able to genotype parental individuals regarding inversions, parental individuals were combined with data from Mykhailenko et al. (2024; 240 individuals). First, parent sequence data was mapped to the *I. typographus* reference genome using Bowtie 2 version 2.4.2 and alignment sorting and indexing was done using Samtools 1.1. Picard MarkDuplicates was used to remove duplicate reads (https://broadinstitute.github.io/picard/) and GATK HaplotypeCaller was used to obtain GVCFs for parents. The datasets (parents and 240 individuals) were combined and genotyped at the stage of GVCFs using GATK modules (CombineGVCFs and GenotypeGVCFs). Variant Quality Score Recalibration (VQSR) and data filtering were performed in the same way as described in Mykhailenko et al. (2024). Shortly, variant quality score log-odds (VQSLOD) were calculated using GATK VariantRecalibrator and ApplyVQSR with the –truth-sensitivity-filter-level set to 99.0. Indels together with polymorphic sites 5 bases up- and downstream from them were removed using Bcftools version 1.11. We applied GATK hard filtering recommendations (GATK Team). We also filtered out variants with MAF < 0.01 and masked all genotypes with low sequencing depth (DP < 8) or low genotype quality (GQ < 20). In addition, variants located within annotated repeated regions of the genome, variants with excessive overall coverage (mean + 1 SD), and variants that exhibited significant heterozygote excess (ExcessHet > 54.69, phred scaled) were masked. Only biallelic sites were kept for downstream analysis.

### Linkage map construction and anchoring

To construct the linkage map, we used Lep-MAP3 (Rastas 2017). The data was converted to likelihoods using Pileup2Likelihood module using default settings. To check if observed relatedness among family members matched the expectations, we used 5% of markers and the IBD module from LepMAp3. We used the ParentalCall module to call informative parental markers (removeNonInformative = 1) using pedigree information for both families. We filtered out markers with MAF < 0.1, high segregation distortion (dataTolerance = 0.01; value recommended when multiple families are used), those with more than 10% of missing genotypes (missingLimit = 0.1), and used makers informative in both families (familyInformativeLimit = 2). To select LOD score threshold and assign markers to linkage groups (LGs), we ran the SeperateChromosomes2 module with different values of parameter lodLimit (ranging from 10 to 27; expected number of chromosomes [2n = 14 + Xy, Virkki 1960]). This procedure computes all pairwise LOD scores between markers and joins markers with LOD score higher than a given lodLimit parameter. We evaluated a range of LodLimit parameters and decided to use LOD = 19 that resulted in 16 large LGs (746 to 88,110 markers). This is one more than expected based on previous karyotyping; however, different LOD scores produced substantially different results—LOD18 yielded only 6 LGs, while LOD20 produced 17. Therefore, we reevaluated the karyotype (see below). We identified sex-linked LG based on the placement of IpsContig9 that consistently exhibited half of the coverage in males in our previous population level re-sequencing study (Mykhailenko et al. 2024). The module OrderMarkers2 was used to order markers within each LG by maximizing the likelihood of the data given the order. We ran the ordering for autosomal (using informativeMask = 123, sexAveraged = 1) and sex-linked LG (using informativeMask = 2) with options outputPhasedData = 1, numMergeIterations = 60, and useKosambi = 1 to correct for crossover interference and multiple recombination events per LG.

To anchor the genome assembly into chromosomes and detect erroneously assembled contigs, we used Lep-Anchor (Rastas 2020). We used information on intervals of genetic marker positions determined by OrderMarkers2 and supported anchoring by PacBio reads produced for the genome assembly (Powell et al. 2021). The reads were mapped to the reference genome using minimap2 (Li 2018). The results were used to produce Marey maps that were investigated to find potential genome assembly errors. In a few contigs, large differences in genetic distance between adjacent markers were found and these contigs were allowed to be cut in such places using the -e option in lepanchor_wrapper2.sh script. This procedure produced a final version of the assembly composed of ordered and cut contigs (IpsContigs). Finally, the physical marker order after the anchoring was used to clean and evaluate the map in the additional OrderMarkers2 runs. To evaluate the influence of polymorphic inversions on recombination across LGs, we ran OrderMarkers2 under several configurations using different families and marker subsets. Specifically, we generated (i) a sex-averaged map using both families; (ii) family-specific, sex-averaged maps using each family independently; and (iii) individual-based (parent-specific) maps. The parent-specific maps were constructed by analyzing markers from one family at a time and using informativeMask = 13 for males and informativeMask = 12 for females. The final Marey maps were plotted using custom R scripts.

### Transposable element and gene annotation in the new assembly

The new assembly was used to detect and classify repeat elements within the genome using EarlGrey (Baril et al. 2024). The initial mask of known transposable elements (TEs) was done using term 7041, which corresponds to the ID number for the order Coleoptera (NCBI taxonomy ID). To characterize TE distribution across the genome, the number of TE bases was summarized in 50 kb windows. Gene annotations from the previous assembly were transferred to the new coordinates using Liftoff v1.6.3 (Shumate and Salzberg 2021). Gene and repeat densities were likewise summarized in 50 kb windows, and enrichment of inversions for genes, repeats, and TEs was assessed using permutation tests.

### Recombination rate and the number of recombination events

Local recombination rates were estimated using a sliding window approach with MareyMapOnline (Siberchicot et al. 2017). In this approach, local recombination rates are estimated using linear regression and an automatically calculated window size that is fixed for each LG. This analysis was performed using all produced maps. Recombination rates (cM/Mb) were plotted against physical distance using custom R scripts. Local recombination rates in collinear regions of the genome were compared with those inside inversions using permutations. For this analysis, we divided the genome into effectively collinear regions (those free of inversions and those where individuals were inversion homozygotes) and inversions where recombination suppression was expected (inversion heterozygotes), defined separately for each parent-specific map. We then calculated the recombination rate (cM/Mb) for every region and compared the mean recombination rates between the 2 groups. To assess significance, we performed 1,000 permutations in which region labels (collinear and inversions) were randomly reassigned.

To test whether inversion genotypes affect the observed recombination rates, we ran OrderMarkers2 with outputPhasedData = 1 and used informativeMask = 1 for males and informativeMask = 2 for females. We then used custom Perl scripts to count the number of observed recombination events based on switches in the paternal or maternal haplotypes. Finally, we tested if we observed significantly fewer recombination events in inversions if individuals were inversion heterozygotes compared to inversion homozygotes (Wilcoxon's rank-sum test; see Supplementary Table 1). Both inversion homozygotes were treated as one group, and recombination event counts were weighted by the number of offspring produced by each parent prior to the test. This means that for each inversion and each parent, the observed number of recombination events was divided by either 65 (family F1) or 82 (family F2).

### Using improved assembly to validate inversions

The new version of genome assembly was used to improve the previously reported description of inversion landscape (Mykhailenko et al. 2024). We used short-read Illumina sequencing data from Mykhailenko et al. (2024) (240 individuals from 18 populations) and mapped reads to the new chromosome-level assembly using Bowtie2 (Langmead and Salzberg 2012) following data processing and filtering steps as in Mykhailenko et al. (2024).

Filtered vcf files were used in downstream analysis to (i) check if the new assembly revealed previously missed putative inversions, (ii) check if the new assembly changed sizes of previously described inversions (e.g. inversions extend through adjacent contigs), and (iii) investigate if previously reported and difficult to interpret variation and linkage disequilibrium (LD) patterns resulted from errors in the previous version of the genome assembly (Mykhailenko et al. 2024). The latter included (i) potential double crossover events identified within 4 inversions (Inv5, Inv18, Inv22.3, Inv22.4); ii) overlapping inversions (Inv7.1 and Inv7.2; Inv16.1 and Inv16.2; Inv23.1 and Inv23.2; 14.6 overlapping with Inv14.1 and 14.2 as well as Inv22.5 overlapping with multiple inversions—Inv22.1 to Inv22.4); and (iii) difficult to disentangle patterns associated with Inv2, Inv5, and Inv7.2. Putative Inv2 showed ambiguous PCA clustering consistent with inversion polymorphisms only when a subset of individuals was analyzed. PCA of Inv5 identified several clusters that could not be associated with inversion genotypes but potentially indicate multiple independent recombination events. Inv7.2 showed an unexpected pattern of low LD in the middle of the inversion and unexpected clustering of individuals in one of the clusters along PC1 with individuals clustered according to their geographic location within the inversion genotype groups. To address the above points 1 to 3, we used population data mapped to the new assembly and (i) calculated LD among SNPs (thinned by selecting one SNP every 10 kb; MAF > 0.2) using PLINK (Purcell et al. 2007), (ii) calculated Fst between individuals previously genotyped as alternative homozygous using VCFtools version 0.1.16 (100 kb overlapping—20 kb—windows (Danecek et al. 2011), and (iii) performed PCA analysis of regions with high LD and/or elevated Fst between homozygotes. The updated inversion boundaries were estimated based on Fst values calculated between inversion haplotypes; we defined the start of the inversion as the point where Fst rose above 0.15 across multiple consecutive windows and the end where it returned to background levels (Fst < 0.15) across adjacent window. The new inversion boundaries represent the region of sequence differentiation between inversion haplotypes rather than the exact inversion breakpoints.

### Nanopore sequencing and inversion breakpoint detection

To confirm the presence of inversions and identify inversion breakpoints, we sequenced 2 pools of 10 *I. typographus* individuals using Oxford Nanopore Technology (ont). Pooling individuals allowed us to obtain more high-quality DNA that is preferable for long-read sequencing. Each pool was sequenced to the approximate coverage of 85×, and the raw data was subject to nanopore-specific processing. Porechop ABI (Bonenfant et al. 2023) was used to trim adaptors, and *chopper* (from Nanopack; De Coster et al. 2018) was used to filter low-quality reads (q < 10), short reads (<100 bp), and reads with not expected GC content (>0.38 and <0.30). NanoPlot was used to summarize the sequencing results before and after trimming and filtering. Minimap2 (Li 2018) was used to map long ont reads to the new chromosome-level assembly. Sniffles2 (Smolka et al. 2024) and cuteSV (Jiang et al. 2020) were used for inversion breakpoint identification. Sniffles2 was used with default options and cuteSV with –max_size -1 –min_support 10 –genotype.

### Karyotyping

Since the results from linkage map reconstruction suggested a different number of chromosomes than previously reported (Virkki 1960), we decided to re-assess *I. typographus* karyotype using classical cytological methods. Young adult specimens were collected from under spruce bark and fixed in a 3:1 ethanol:glacial acetic acid solution. Slides for cytogenetic studies were prepared by the squash method. Testes were extracted from the abdomen in a drop of 45% acetic acid and squashed under cover glasses, which were removed by a dry ice technique, and the slides were air dried. The best slides were stained using a standard Feulgen–Giemsa procedure (Grozeva and Nokkala 1996). They were hydrolyzed in 1 N HCI at room temperature for 20 min, then in 1 N HCl at 60 °C for 8 min, and stained in Schiff's reagent for 20 min. After rinsing in distilled water, the slides were stained in 2% Giemsa in Sorensen's buffer pH 6.8 for 1 min, rinsed briefly in distilled water, air dried, and mounted in Entellan. The preparations were examined under a Leica MM 4000 microscope at l000×, and photomicrographs were taken with a Nikon DS-U I camera.

### Comparison to *Ips nitidus* genome

To investigate the synteny of *I. typographus* genome with its closest relative, *I. nitidus*, we compared the new genome assembly to the chromosome-level assembly of *I. nitidus* (NCBI: GCA_018691245.2; 1.5–2.3 Mya divergence from the spruce bark beetle) (Wang et al. 2023). In both species, we focus only on the part of the genome assemblies that were assigned to chromosomes (16 chromosomes in *I. nitidus* and 15 LGs plus putative Y chromosome in *I. typographus;* see details below). Syntenic blocks were detected using ntSynt version 1.0.1 (Coombe et al. 2024). The results were visualized using gggenomes version 1.0.0 in R (https://github.com/thackl/gggenomes) and following ntSynt manual.

## Results

### Sequencing, linkage mapping, and karyotyping

Our mapping population consisted of 151 individuals coming from 2 families (65 and 82 offspring). Parents were sequenced to a high coverage (mean 176×) while offspring were initially sequenced to varying coverage, but their final coverage was standardized to ca. 20×. After this procedure and removal of duplicate reads, the data used for downstream analysis had an average coverage of 15.6× (standard deviation: 0.85) for offspring and 15.8× (0.50) for parents. The presented linkage maps are based on the updated genome assembly described in the next section.

Following the Lep-MAP3 pipeline for whole-genome re-sequencing data, we obtained 2,004,155 markers after ParentCall and 506,123 that were assigned to LGs after all the applied filters (such a reduction in number of SNPs is common after filtering steps in Lep-MAP3). The majority of these markers (506,087) were assigned to the 16 largest LGs, likely representing 16 chromosomes (15 autosomes and an X chromosome). Linkage group 16 (LG16) included IpsContig9 previously identified as a part of the X chromosome (Mykhailenko et al. 2024) and thus was treated as a sex chromosome. Since the identified number of LGs was different from the previously reported number of *I. typographus* chromosomes, we reevaluated the karyotype with cytogenetic methods (Supplementary Fig. 1). Karyotyping clearly indicated that the spruce bark beetle carries 15 autosomal pairs and a Xy pair of sex chromosomes, consistent with 16 LGs detected here.

**Fig. 1.**
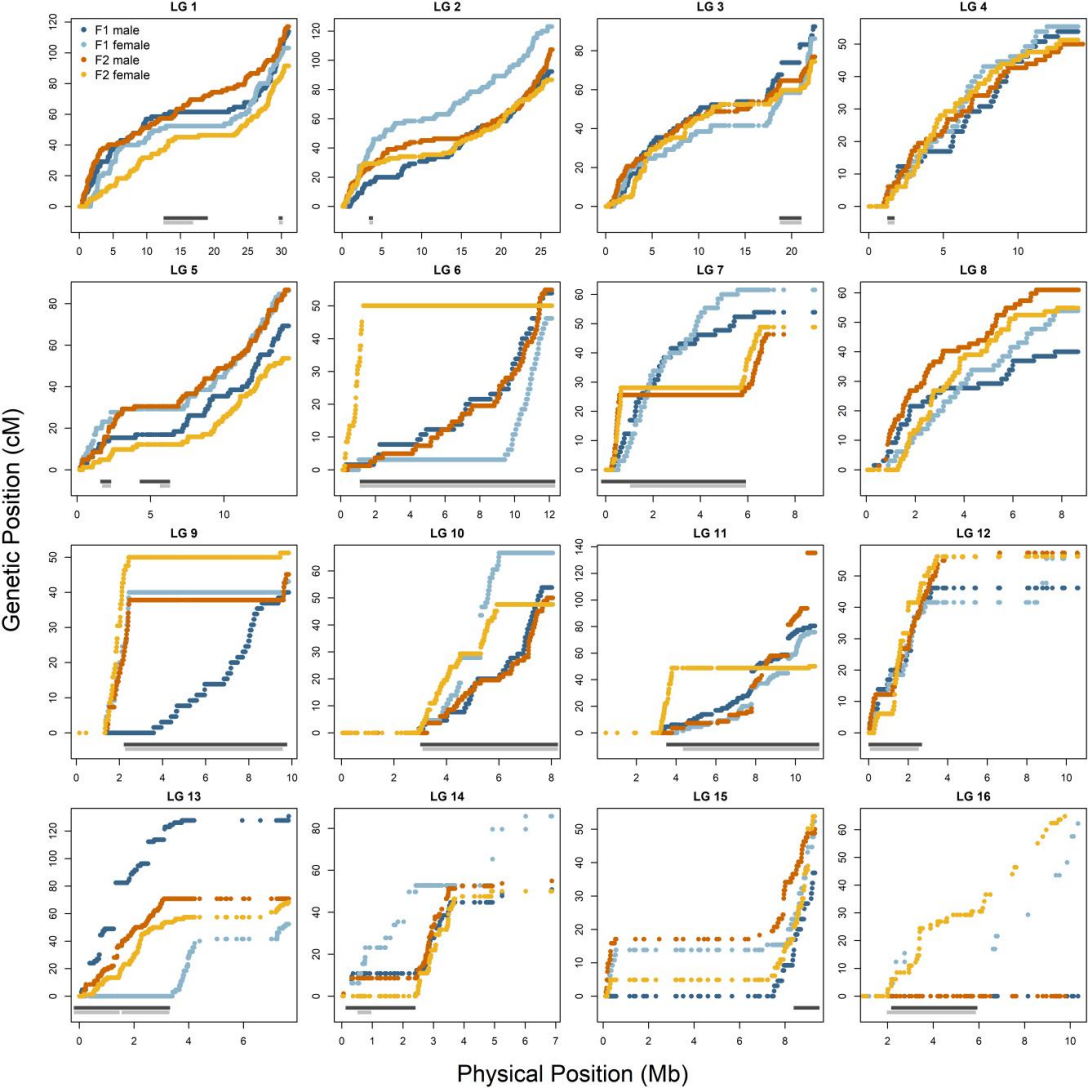
The Eurasian spruce bark beetle Marey map. Parent-specific genetic distance (cM) on the *y* axis against the physical distance (Mb) on the *x* axis for 16 LGs likely corresponding to the bark beetle chromosomes. LG1-LG15, autosomes; LG16, X chromosome. Light gray rectangles indicate the position of polymorphic inversions based on the Mykhailenko et al. (2024), and dark gray rectangles indicate the inversion coordinates as updated in the current study. Information on parents is included as an inset in LG1. Blue colors indicate family F1 and yellow/orange family F2.

The total length and number of markers in each of the LGs for all reconstructed maps are presented in Table 1 and Supplementary Tables 2 and 3. The total length of the autosomal, sex-, and family-averaged map equaled 978 cM (467,154 markers in total after genome anchoring; 1,040 cM and 467,278 markers with the sex chromosome). LG sizes ranged from 45 to 116 cM and the average physical distance between markers equaled 449 bp. The total male and female autosomal map lengths (family averaged) were similar and spanned 1,004 cM (408,537 markers) in males and 954 cM (337,820 markers; 1,017 cM and 337,696 including the sex chromosome) in females. The total autosomal map lengths for other maps (parent-specific and family-specific) ranged from 888 to 1,064 cM (from 259,687 to 445,159 markers; Supplementary Tables 2 and 3). The final Marey maps of each LGs show an expected steady increase in genetic distance with physical position (Fig. 1; Supplementary Figs. 2 to 6).

**Table 1.** The total length and number of markers in each LG for male, female, and sex-averaged map reconstructed using both families.

	Male		Female		Sex averaged		
LG	Length (cM)	Markers	Length (cM)	Markers	Length (cM)	Markers	Length (Mb)
1	115.65	76,527	97.68	69,705	106.66	88,081	31.12
2	99.33	61,538	102.73	51,305	101.02	68,683	26.55
3	83.69	43,396	79.60	34,497	81.64	48,256	22.52
4	51.70	42,290	53.06	34,587	52.38	47,054	14.41
5	78.92	31,249	68.03	27,964	73.47	36,991	14.45
6	54.43	31,239	48.31	21,752	51.36	32,153	12.14
7	49.66	29,787	54.43	12,439	52.04	31,548	9.59
8	51.71	24,911	54.43	19,924	53.06	27,783	8.70
9	42.86	25,153	47.63	20,786	45.24	25,960	9.86
10	51.71	11,370	55.83	22,135	53.75	24,595	8.07
11	99.83	12,940	59.90	9,503	81.1	15,728	11.01
12	52.39	9,642	49.68	5,519	51.02	10,256	10.60
13	75.81	4,161	61.23	3,780	67.38	5,233	7.77
14	52.45	2,693	65.40	2,374	58.86	3,024	7.08
15	44.23	1,641	56.36	1,426	48.65	1,809	9.53
16			63.35	124	63.35	124	9.65

The sex- and family-averaged map reflected recombination patterns averaged across individuals with different inversion genotypes. Although inversion effects are already visible in family-specific maps (Supplementary Fig. 6), they become much clearer in the parent-specific maps (Fig. 1; also visible at parent-specific recombination rates [cM/Mb] plotted along LGs, Supplementary Fig. 7). In many inversions, particularly the larger ones, complete suppression of recombination is evident in individuals genotyped as inversion heterozygotes (e.g. Inv13 on LG7, where the 2 heterozygous individuals showed drastic suppression of recombination compared to homozygous individuals, which exhibited a steady increase in genetic distance along the LG). Parent-specific maps also revealed that some inversions may produce unexpected or difficult-to-interpret patterns, potentially due to additional structural variation within the inverted segments. For example, the inversion on LG13 shows the expected pattern of recombination suppression in the heterozygous female, normal recombination in 2 homozygous parents for 1 inversion haplotype, but a disrupted recombination pattern in the male carrying the alternative inversion haplotype—suggesting additional structural complexity in this individual (Inv18). Nanopore reads further support this interpretation, indicating 2 possible rearrangements in the same region (Table 2; Supplementary Fig. 8). A similar pattern is observed on LG11, with the expected suppression in the inversion heterozygote but qualitatively different recombination patterns in the 2 alternative inversion homozygotes. This approach also highlights potential additional structuring in other inversions, such as LG6 (Inv5).

**Table 2.** A list of identified inversions, including their genomic coordinates and available information on inversion breakpoints.

LG	Inversion	Start	End	Size (Mb)	Nanopore support	Additional information
LG1	Inv2	12,700,001	18,880,001	6.16	Partial	Inversion region larger compared to previous estimates due to the joining to other contigs, multiple inversion breakpoints within the region
LG1	Inv3	29,780,001	29,980,001	0.18	No	
LG2	Inv12	3,560,001	3,680,001	0.1	Yes	Nanopore inversion coordinates: 3,629,475–3,703,006
LG3	Inv15	18,860,001	20,860,001	1.98	No	
LG4	Inv10	1,380,001	1,540,001	0.14	Yes	Nanopore inversion coordinates: 1,464,549–1,548,661
LG5	Inv17	1,780,001	2,140,001	0.34	No	
LG5	Inv6	4,460,001	6,160,001	1.68	Yes	Inversion region larger compared to previous estimates due to joining to other contig and some rearrangements; nanopore inversion coordinates: 4,850,890–6,334,125
LG6	Inv5	1,220,001	12,144,430	10.92	No	
**LG7**	**InvLG7**	**1**	**660,001**	**0.66**	**No**	**New inversion based on LD and updated assembly**
LG7	Inv13	580,001	5,720,001	5.12	Yes	Inversion region larger compared to previous estimates due to the joining to other contigs; nanopore inversion coordinates: 1,440,191–5,720,319
LG9	Inv7.1	8,640,001	9,320,001	0.66	Yes	Nanopore inversion coordinates: 8,713,192–9,320,169
LG9	Inv7.2	2,400,001	9,580,001	7.16	No	
LG10	Inv14.1	5,920,001	8,020,001	2.08	Yes	Nanopore inversion coordinates: 6,000,931–7,283,706
LG10	Inv14.2	5,280,001	7,960,001	2.66	No	Inversion coordinates different from the previously reported and now based on Fst
LG10	Inv14.3	4,460,001	5,300,001	0.82	No	
LG10	Inv14.4	4,160,001	4,320,001	0.14	Partial	Two pairs of inversion breakpoints identified in the region
LG10	Inv14.5	3,200,001	3,820,001	0.62	No	
LG10	Inv14.6	5,640,001	8,074,229	2.43	No	
LG11	Inv16.2 + 23.2	3,700,001	10,680,001	6.94	Partial	Multiple shorter inversions identified with nanopore sequencing within the region
LG11	Inv16.1+23.1	3,700,001	11,013,495	7.31	Partial	Multiple shorter inversions identified with nanopore sequencing within the region
LG12	Inv22.1	1,960,001	2,500,001	0.52	Yes	Nanopore inversion coordinates: 2,023,103–2,491,709
LG12	Inv22.2	1,680,001	2,000,001	0.32	No	
LG12	Inv22.3	400,001	1,680,001	1.26	Partial	Multiple shorter inversions identified with nanopore sequencing within the region but consistent with inferred double crossover
LG12	Inv22.4	220,001	440,001	0.22	Yes	Nanopore inversion coordinates: 352,375–442,895
LG12	Inv22.5	180,001	2,340,001	2.14	No	
LG13	Inv18	1	3,120,001	3.12	Partial	Maybe 2 rearrangements; inversion region—bigger compared to previous estimates due to connection to other contig and some rearrangements
LG14	Inv26	240,001	2,320,001	2.06	Yes	Inversion region—bigger compared to previous estimates due to connection to other contig and some rearrangements; nanopore inversion coordinates: 458,781–2,043,057
**LG15**	**InvLG15.1**	**8,580,001**	**8,720,001**	**0.14**	**Partial**	**New inversion based on LD and updated assembly; multiple inversions breakpoints within the region**
**LG15**	**InvLG15.2**	**8,920,001**	**9,400,001**	**0.48**	**No**	**New inversion based on LD and updated assembly**
LG16	Inv9	2,280,001	6,000,001	3.72	No	Inversion coordinates different from the previously reported and now based on Fst

Breakpoint coordinates identified through Nanopore sequencing are listed in the “Additional information” column for the corresponding inversions. All inversions identified with nanopore sequences are provided as vcf file in the Supplementary Information. This column also includes notes for inversions whose estimated sizes differ from those reported in Mykhailenko et al. (2024). Newly identified inversions described in this study are highlighted in bold.

The mean recombination rate for the sex- and family-averaged linkage map equaled 4.9 cM/Mb and ranged from 3.4 to 8.7 cM/Mb among LG (Supplementary Fig. 9). Smaller LGs showed higher recombination rates (Pearson correlation, *r*^2^ = 0.47; *P* = 0.003, Supplementary Fig. 10), and there was also a strong significant correlation between LG genetic length and its physical size (Pearson correlation; *r*^2^ = 0.71; *P* << 0.001; Supplementary Fig. 11). Similar mean recombination rate was estimated for family-specific and parent-specific maps (ranged from 4.2 to 5.0 cM/Mb; Supplementary Figs. 11 and 12). We identified a total of 2,988 recombination events across all LGs, of which 976 occurred within regions harboring putative polymorphic inversions. We observed significantly fewer recombination events within inversions for which parents were heterozygous compared to homozygous parents (Wilcoxon rank-sum test, *P* << 0.0001; Supplementary Fig. 13 and Table 1).

### Chromosome-level genome assembly and updated inversion landscape

The reconstructed sex- and family-averaged linkage map was used to anchor *I. typographus* contigs and obtain a chromosome-level genome assembly. The previous assembly consisted of 272 contigs (237 Mb in total), 140 of them were now assembled into 16 LGs (213 Mb; 90% of the assembly). Fifty-four other contigs are also included as contigs not assigned to chromosomes (12 Mb, 5%), and 83 small contigs were excluded by LeP-anchor from the updated reference (12 Mb, 5%). Repeats constitute more than half of the updated genome (51.4%). LGs LG12 to LG15 had significantly more repeats than the rest of the LGs (Mann–Whitney *U* test; *P* = 0.0005; Supplementary Table 4). TEs comprise a substantial fraction of repeats (23% of the total genome), and the most abundant groups of TEs are LTRs (12.7%), LINEs (4.9%), and DNA transposons (4.1%; Supplementary Fig. 14). SINEs, Penelope, and RC (rolling circle) together cover 1.2% of the genome.

We identified several misassemblies present in the previous version of the *I. typographus* genome. Five small parts (in total 0.7 Mb) of 4 contigs were removed from the contigs that were incorporated into LGs (Supplementary Table 5). Most of the removed sequence (0.5 Mb) belonged to IpsContig35 that behaves like it is sex-linked (see below). Seven contigs were cut and rearranged in different order within the same LG, 3 others were divided into parts that ended up in different LGs, and 1 was elongated by introducing gaps in several places (Supplementary Table 5). Many of these cases were associated with inversions (within inversions or adjacent to them; Supplementary Table 5) confirming that assembly of a genome containing multiple polymorphic inversions can be challenging.

The new assembly led to only modest changes in the previously described inversion landscape. Six putative inversions (Inv2, Inv6, Inv13, Inv16.1 + Inv23.1, Inv18, Inv26) were extended due to the inclusion of additional contigs—either through connections to larger contigs or the addition of smaller ones that were not part of the original inversion analysis by Mykhailenko et al. (2024) (Supplementary Table 3). Two other inversions (Inv9 and Inv14.2) were expanded because their boundaries were redefined based on estimates of Fst between homozygotes (Supplementary Table 6). In most cases, patterns that were previously difficult to interpret (see above) remained challenging to interpret despite improved assembly. For example, Inv2 increased in size due to its connection with IpsContig3, expanding the region of strong LD and elevated Fst between previously identified homozygous groups (Supplementary Fig. 8). However, the inversion-like PCA pattern in this region was only observed when analyzing individuals from southern populations and was restricted to a subregion of the expanded putative inversion. Inv5 retained the same PCA and LD patterns as before, despite rearrangements involving IpsContig5. Similarly, Inv7.2 exhibited an identical PCA and LD pattern to that reported by Mykhailenko et al. (2024). Patterns used to identify potential double crossovers within 4 inversions (Inv5, Inv18, Inv22.3, and Inv22.4) also remained unchanged, despite contigs sequence rearrangements in Inv5 and Inv18 (Supplementary Fig. 8). Overlapping inversion signals—such as Inv7.1 and Inv7.2; Inv16.1 and Inv16.2; Inv23.1 and Inv23.2; Inv14.6 overlapping with Inv14.1 and Inv14.2; and Inv22.5 overlapping with Inv22.1–Inv22.4—produced patterns similar to those previously described. However, the number of recombination events inferred in parents for Inv16.2 + Inv23.2 and Inv22.5 was inconsistent with their genotypes: heterozygous individuals produced a high number of recombinants (Supplementary Table 1). These results suggest that these inversions may not represent independent inversions and that the observed PCA patterns may instead reflect other structural variation (e.g. duplications) or assembly errors.

Additionally, the new LD analysis revealed several regions of elevated LD that had not been identified before (Supplementary Table 7). Three of these regions exhibited inversion-like PCA patterns (i.e. 3 distinct genotype clusters; Supplementary Figs. 8 and 15) and were treated as newly identified inversions.

### Mapping inversion breakpoints with nanopore sequencing

Nanopore sequencing yielded a mean read length of approximately 2,900 bp in both sequenced pools, with N50 read lengths of 5,012 bp and 4,756 bp, respectively. The mean coverage of both pools equaled 55×. Due to limitations in DNA extraction quality and library preparation, it was not possible to identify breakpoints for all inversions. However, we found strong support for 9 polymorphic inversions at positions corresponding to those previously reported by Mykhailenko et al. (2024) and partial support for 7 other inversions (Table 2; Supplementary Fig. 8, in the Supplementary Information). The latter includes cases where multiple inversion breakpoints were detected within a single inversion region, suggesting a more complex genomic architecture—such as overlapping structural variants segregating within the population. In most cases, the sequence differentiation (measured by Fst) of 2 inversion haplotypes clearly extends beyond the potential inversion breakpoints (Supplementary Fig. 8). Updated inversion positions, their sizes, and the information on the nanopore reads support for inversion breakpoints are provided in Table 2.

Linkage mapping and genome anchoring revealed that one of the contigs (IpsContig35, 1.1 Mb long) was divided into 2 parts, one of which (0.6 Mb) was assigned to LG16 (X chromosome) and the other remained separate among contigs not assigned to any of the 16 LGs. This observation led us to investigate whether this part of the contig constitutes Y chromosome in the spruce bark beetle. Mapping of sequencing reads obtained from 240 individuals from 18 European populations (Mykhailenko et al. 2024) to a new genome assembly revealed that the coverage of the putative Y chromosome is similar to the X chromosome in both male individuals (half of the coverage observed at autosomes) and female individuals (coverage similar to the one observed at autosomes). Thus, we could not confirm the discovery of the Y chromosome, and it remains unclear whether this part of IpsContig36 is part of the X chromosome or not.

### Inversions vs collinear genome

**Fig. 2.**
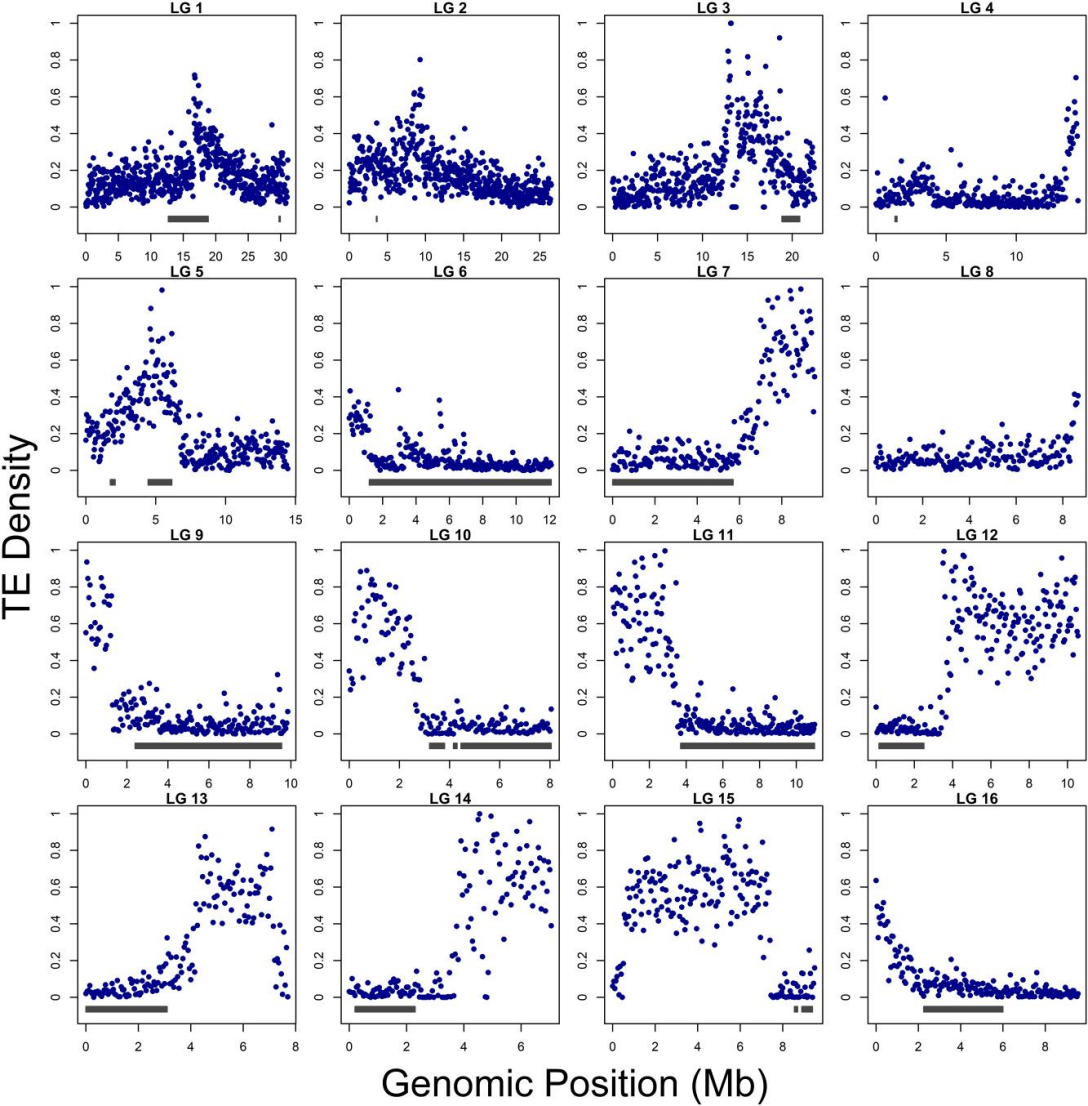
Distribution of TEs across LGs. TE density was quantified in 50 kb sliding windows by calculating the total length of TE sequences within each window divided by the window size, providing a normalized measure of TE abundance along each LG. Gray bars indicate the positions of chromosomal inversions.

The recombination rate was lower in inversions (inversion heterozygotes) compared to collinear parts of the genome (permutation test, *P* < 0.005, collinear mean = 9.54 cM/Mb, and inversion mean = 3.24 cM/Mb). We observed a large variation in recombination rate in collinear and inversion parts across LGs (Supplementary Fig. 16) and number of recombination events inferred in inversions mostly consistent with individuals' genotypes (no or very low recombination counts in heterozygous parents; Supplementary Table 1). In addition, we found that inversions (all genotypes) are depleted in repeats in general (permutation test, *P* < 0.0001; Supplementary Fig. 17), including TEs (permutation test, *P* < 0.0001; Fig. 2), and enriched in genes (Supplementary Fig. 18; permutation test, *P* < 0.0001).

### Comparison with *I. nitidus* genome

**Fig. 3.**
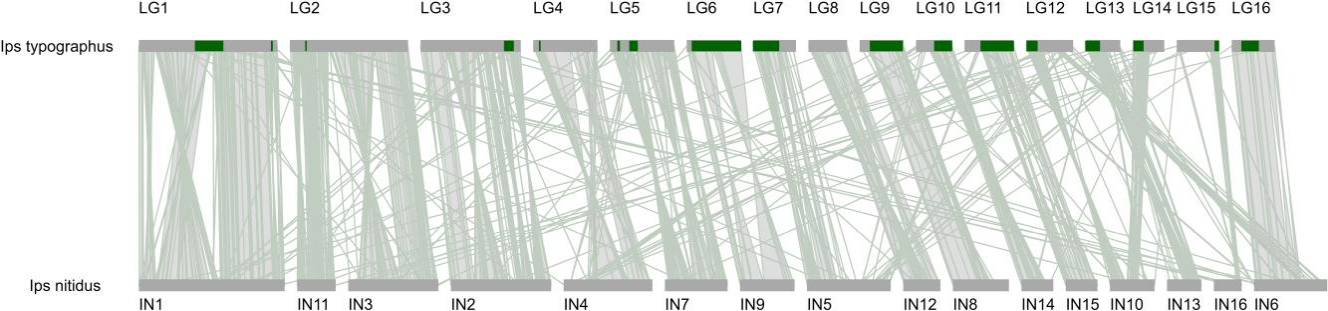
Synteny plot between *I. typographus* LGs (LG1 to LG16) and 16 *I. nitidus* chromosomes (IN1 to IN16). For clarity, orientation of chromosomes IN1, IN4, IN9, and IN10 was reversed to match the *I. typographus* orientation. Polymorphic inversions identified in the *I. typographus* genome are indicated as green bars. Species were characterized by high synteny, but several interchromosomal rearrangements were also observed including at least 2 fission/fusion events (e.g. LG2 and IN11/IN3) and several interspecies inversions (e.g. LG1 and IN1). Least synteny was associated with LGs with the highest proportion of repeats (LG12 to LG15).

We compared the updated genome assembly to the chromosome-level assembly of *I. nitidus*—a closely related species and the only one from the *Ips* genus for which a genome assembly is available. Alignment of the 2 genomes confirmed previously reported high synteny between species (Mykhailenko et al. 2024; Fig. 3). Despite the same number of chromosomes in both species, several interchromosomal rearrangements were observed including at least 2 fission/fusion events and several inversion differences.

## Discussion

Insects exhibit remarkable diversity in recombination rates, ranging from over 20 cM/Mb in some social insects to less than 1 cM/Mb in several dipteran species (Wilfert et al. 2007; Stapley et al. 2017a; Stapley et al. 2017b; DeLory et al. 2024). The total family and sex- averaged genetic map length and average recombination rate in the spruce bark beetle (*I. typographus*), 1,040 cM and 4.89 cM/Mb, respectively, fall well within this range and are comparable to those reported for the other bark beetle *Dendroctonus ponderosae*, which has a map length of 1,069 cM and a recombination density of approximately 4.77 cM/Mb (Keeling et al. 2022; Pushman et al. 2025). Similarly, the updated estimates of repeat and TE content in *I. typographus* are consistent with patterns observed in other insect genomes. Repetitive sequences constitute approximately 50% of the genome, a substantial increase from the previously reported 28% (Powell et al. 2021). This revised estimate aligns closely with values observed in related species, including *I. nitidus* (46%; Wang et al. 2023), *Dendroctonus valens* (45%; Liu et al. 2022), and *Euwallacea fornicatus* (47%; Bickerstaff et al. 2024). TEs alone comprise at least 23% of the genome, with LTR, LINE, and DNA transposons being the most abundant. This TE composition is broadly similar to that reported for *I. nitidus* (Wang et al. 2023).

As expected, the recombination landscape in *I. typographus* is strongly influenced by inversion polymorphisms. Individuals heterozygous for inversions (i.e. parents carrying one inverted and one standard arrangement) exhibit a significant reduction in recombination within the inverted region, often showing complete recombination suppression, compared to homozygous individuals at the same region. Recombination is spatially variable across the genome and varies among individuals depending on their inversion genotypes. Given the high number and extensive genomic coverage of polymorphic inversions (nearly 30% of the genome) and their frequencies, it is likely that many individuals in nature are heterozygous for at least some inversions. Consequently, recombination rates are generally lower in inversions than in collinear regions, as confirmed by our findings.

Interestingly, we found that inversions are depleted in TEs. While TEs are known to facilitate inversion formation (Gray 2000; Lee et al. 2008; Puig et al. 2015; Huang and Rieseberg 2020; Berdan et al. 2023) and can accumulate—alongside other weakly deleterious mutations—within inversions due to reduced purging in regions of suppressed recombination (Jay et al. 2021; Berdan et al. 2023), our results indicate a lack of TE enrichment. This observation aligns with our previous analysis, which found no evidence for increased mutational load within species inversions (Mykhailenko et al. 2024).

In contrast, inversions appear to be enriched for genes. Growing evidence suggests that polymorphic inversions are rarely selectively neutral (Berdan et al. 2023), often facilitating local adaptation by preserving advantageous combinations of alleles from different genes in the face of gene flow (Kirkpatrick and Barton 2006; Harringmeyer and Hoekstra 2022). This may also hold true for *I. typographus*, where inversions may be associated with fitness-related traits, including genes involved in odorant perception (Mykhailenko et al. 2024). The improved genome assembly and recombination map presented here offer a valuable resource for future functional studies. These include investigations into olfactory gene polymorphisms across the species' range and their potential linkage to inversion polymorphisms (Johny et al. 2025), as well as efforts to disentangle the evolutionary forces shaping genome variation and maintaining inversion polymorphisms in this ecologically important species.

Finally, the identification of several misassemblies in the previous genome assembly underscores the challenges of assembling genomes with extensive structural polymorphisms. Despite these difficulties, the updated assembly remains largely consistent with most previously described inversion patterns. Notably, our incorporation of Nanopore long-read sequencing not only provided direct support for many inversions but also facilitated the identification of putative inversion breakpoints. However, certain regions, particularly those involving overlapping inversions or complex recombination patterns, such as double-crossover events, remain difficult to resolve with high confidence. For example, 2 overlapping inversions (Inv14.6 and Inv22.5) appear to represent either assembly artifacts or alternative structural variants, as they exhibit recombination patterns in heterozygous individuals that contradict the expected suppression of recombination typically observed in such regions. In addition, our analysis revealed that the karyotype of *I. typographus* includes one more pair of autosomal chromosomes than previously reported. This finding highlights the challenges of accurate karyotyping in some species and demonstrates how linkage mapping can provide a valuable framework for refining chromosome number and structure.

## Conclusions

The genetic map of *I. typographus* enabled the construction of a chromosome-level genome assembly and provided crucial genomic resources for the most species-rich yet underrepresented with respect to genomic information, insect orders—Coleoptera (Hotaling, Kelley, et al. 2021a; Hotaling, Sproul, et al. 2021b). The updated assembly with updated repeat content and composition and species-specific recombination map lays the foundation for in-depth investigations into the adaptation genomics of *I. typographus* and serves as a valuable reference for comparative genomic studies across beetles and other insect groups. Importantly, we further supported the existence of complex inversion polymorphic landscape in the Eurasian spruce bark beetle and showed how much recombination is suppressed in inversions compared to the collinear parts. The spruce bark beetle inversions are strong recombination modifiers of regions enriched in genes potentially playing a role in species adaptation.

## Supplementary Material

jkag017_Supplementary_Data

## Data Availability

Sequencing data are deposited at the National Center for Biotechnology Information Sequence Read Archive under the updated BioProject ID PRJNA1313600. The genome assembly produced in this study has been deposited in NCBI at DDBJ/ENA/GenBank under the accession JADDUH000000000. The version described in this paper is version JADDUH020000000. Additional files and linkage mapping pipeline have been deposited in Figshare at https://doi.org/10.6084/m9.figshare.31028410. Supplemental material available at G3 online.
