# Supplementary material for: Recombination landscape shaped by inversion polymorphisms: a high-density linkage map and chromosome-level assembly of inversion-rich spruce bark beetle genome
Source: G3 (Bethesda). 2026 Jan 20;16(4):jkag017. doi: 10.1093/g3journal/jkag017 (PMC13042298; doi:10.1093/g3journal/jkag017)
Supplement: jkag017_Supplementary_Data [file jkag017_supplementary_data.docx]

**Supplementary Information**

Recombination landscape shaped by inversion polymorphisms – a high-density linkage map and chromosome level assembly of inversion-rich spruce bark beetle

Krystyna Nadachowska-Brzyska, Anna Maryańska-Nadachowska, Dineshkumar Kandasamy, Martin N. Andersson, Zuzanna Nowak, Piotr Zieliński, Matias Rodriguez, Wiesław Babik

**Table S1** Parent inversion genotypes and inferred number of recombination events. LG – linkage group; F1, F2 – family IDs; F – female; M- male. Recombination event counts represent the total number of inferred crossover events, estimated by counting transitions between phased maternal or paternal haplotypes across markers. All heterozygotes are shown in pink and their corresponding recombination counts are yellow.

|  |  | Inversion genotypes | | | | Recombination events counts | | | |
| --- | --- | --- | --- | --- | --- | --- | --- | --- | --- |
| LG | Inversion ID | F1F | F1M | F2F | F2M | F1F | F1M | F2F | F2M |
| LG1 | Inv2 | AB | AA | AA | AA | 1 | 1 | 7 | 12 |
| LG1 | Inv3 | BB | AB | BB | AB | 0 | 0 | 0 | 3 |
| LG2 | Inv12 | AA | AA | AA | AA | 0 | 0 | 0 | 1 |
| LG3 | Inv15 | AB | BB | AB | AB | 1 | 9 | 0 | 0 |
| LG4 | Inv10 | BB | AA | AA | AB | 0 | 1 | 0 | 0 |
| LG5 | Inv17 | AB | AA | AB | AA | 0 | 1 | 0 | 5 |
| LG5 | Inv6 | AA | AA | AA | AA | 0 | 0 | 0 | 0 |
| LG6 | Inv5 | AA | AA | AB | AA | 27 | 34 | 0 | 45 |
| LG7 | Inv13 | AA | AA | AB | AB | 38 | 29 | 0 | 0 |
| LG7 | InvLG7 | BB | BB | BB | BB | 2 | 4 | 16 | 20 |
| LG9 | Inv7.1 | AB | AB | BB | AB | 0 | 0 | 0 | 0 |
| LG9 | Inv7.2 | AB | BB | AB | AB | 7 | 25 | 0 | 4 |
| LG10 | Inv14.1 | AB | AA | AB | AA | 4 | 22 | 2 | 25 |
| LG10 | Inv14.2 | BB | AB | BB | AB | 15 | 22 | 15 | 26 |
| LG10 | Inv14.3 | BB | AA | AB | AA | 7 | 13 | 2 | 0 |
| LG10 | Inv14.4 | AB | AB | AA | BB | 1 | 0 | 0 | 1 |
| LG10 | Inv14.5 | AA | AB | BB | AA | 4 | 1 | 11 | 5 |
| LG10 | Inv14.6 | AB | AA | AB | AA | 4 | 22 | 0 | 25 |
| LG11 | Inv16.1+23.1 | AA | AA | AB | BB | 49 | 47 | 1 | 81 |
| LG11 | Inv16.2+23.2 | AA | AA | AB | BB | 49 | 46 | 10 | 81 |
| LG12 | Inv22.1 | BB | BB | AB | BB | 6 | 7 | 2 | 9 |
| LG12 | Inv22.2 | AA | AA | BB | AB | 6 | 4 | 2 | 2 |
| LG12 | Inv22.3 | AB | AB | AA | AA | 8 | 8 | 20 | 20 |
| LG12 | Inv22.4 | AB | AB | BB | AA | 3 | 1 | 4 | 3 |
| LG12 | Inv22.5 | AB | AB | BB | BB | 23 | 21 | 34 | 24 |
| LG13 | Inv18 | AB | BB | AA | AA | 0 | 59 | 43 | 58 |
| LG14 | Inv26 | AA | AB | AB | AB | 19 | 2 | 0 | 0 |
| LG15 | InvLG15.1 | AA | BB | AB | BB | 4 | 2 | 2 | 2 |
| LG15 | InvLG15.2 | AA | AA | AA | AB | 9 | 6 | 14 | 0 |
| LG16 | Inv9 | BB | BB | BB | BB | 5 | NA | 24 | NA |

**Table S2** The total length and number of markers in each of the linkage group for parent-specific maps.

| **LG** | **Family** | **Parent** | **Length (cM)** | **Markers** | **Recombination rate (cM/Mb)** |
| --- | --- | --- | --- | --- | --- |
| 1 | F1 | FEMALE | 103.1 | 59330 | 3.31 |
| 2 | F1 | FEMALE | 123.1 | 42157 | 4.64 |
| 3 | F1 | FEMALE | 86.2 | 29328 | 3.83 |
| 4 | F1 | FEMALE | 55.4 | 28953 | 3.84 |
| 5 | F1 | FEMALE | 86.2 | 23379 | 5.96 |
| 6 | F1 | FEMALE | 46.2 | 19719 | 3.80 |
| 7 | F1 | FEMALE | 61.6 | 9689 | 6.42 |
| 8 | F1 | FEMALE | 53.9 | 16309 | 6.19 |
| 9 | F1 | FEMALE | 43.1 | 16401 | 4.37 |
| 10 | F1 | FEMALE | 66.7 | 20466 | 8.26 |
| 11 | F1 | FEMALE | 75.8 | 7504 | 6.89 |
| 12 | F1 | FEMALE | 57.1 | 4441 | 5.39 |
| 13 | F1 | FEMALE | 52.4 | 3440 | 6.74 |
| 14 | F1 | FEMALE | 85.8 | 2033 | 12.12 |
| 15 | F1 | FEMALE | 53.9 | 1274 | 5.66 |
| 16 | F1 | FEMALE | 62.2 | 28 | 6.44 |
| 1 | F1 | MALE | 113.9 | 55545 | 3.66 |
| 2 | F1 | MALE | 92.3 | 42087 | 3.48 |
| 3 | F1 | MALE | 92.4 | 22742 | 4.10 |
| 4 | F1 | MALE | 53.9 | 27048 | 3.74 |
| 5 | F1 | MALE | 69.3 | 20637 | 4.79 |
| 6 | F1 | MALE | 53.9 | 18959 | 4.44 |
| 7 | F1 | MALE | 53.9 | 11857 | 5.62 |
| 8 | F1 | MALE | 40.0 | 14361 | 4.60 |
| 9 | F1 | MALE | 40.0 | 16320 | 4.06 |
| 10 | F1 | MALE | 53.9 | 8410 | 6.67 |
| 11 | F1 | MALE | 80.5 | 7331 | 7.31 |
| 12 | F1 | MALE | 46.2 | 4884 | 4.36 |
| 13 | F1 | MALE | 130.7 | 1435 | 16.83 |
| 14 | F1 | MALE | 50.9 | 1949 | 7.18 |
| 15 | F1 | MALE | 36.9 | 1122 | 3.88 |
| 1 | F2 | FEMALE | 91.5 | 56877 | 2.94 |
| 2 | F2 | FEMALE | 86.6 | 42122 | 3.26 |
| 3 | F2 | FEMALE | 74.4 | 26215 | 3.30 |
| 4 | F2 | FEMALE | 51.2 | 27226 | 3.55 |
| 5 | F2 | FEMALE | 53.7 | 22643 | 3.71 |
| 6 | F2 | FEMALE | 50.0 | 15665 | 4.12 |
| 7 | F2 | FEMALE | 48.8 | 9407 | 5.09 |
| 8 | F2 | FEMALE | 54.9 | 15843 | 6.31 |
| 9 | F2 | FEMALE | 51.2 | 15212 | 5.20 |
| 10 | F2 | FEMALE | 47.6 | 20477 | 5.89 |
| 11 | F2 | FEMALE | 50.0 | 8751 | 4.54 |
| 12 | F2 | FEMALE | 56.2 | 4265 | 5.30 |
| 13 | F2 | FEMALE | 68.3 | 2865 | 8.79 |
| 14 | F2 | FEMALE | 50.0 | 1838 | 7.07 |
| 15 | F2 | FEMALE | 53.9 | 1193 | 5.65 |
| 16 | F2 | FEMALE | 64.8 | 128 | 6.72 |
| 1 | F2 | MALE | 117.1 | 59729 | 3.76 |
| 2 | F2 | MALE | 107.3 | 48990 | 4.04 |
| 3 | F2 | MALE | 76.8 | 33951 | 3.41 |
| 4 | F2 | MALE | 50.0 | 33235 | 3.47 |
| 5 | F2 | MALE | 86.6 | 25353 | 5.99 |
| 6 | F2 | MALE | 54.9 | 23899 | 4.52 |
| 7 | F2 | MALE | 46.4 | 23797 | 4.84 |
| 8 | F2 | MALE | 61.0 | 18633 | 7.01 |
| 9 | F2 | MALE | 45.1 | 23175 | 4.58 |
| 10 | F2 | MALE | 50.0 | 8329 | 6.19 |
| 11 | F2 | MALE | 135.4 | 7072 | 12.30 |
| 12 | F2 | MALE | 57.3 | 8203 | 5.41 |
| 13 | F2 | MALE | 70.8 | 3398 | 9.11 |
| 14 | F2 | MALE | 55.0 | 2244 | 7.76 |
| 15 | F2 | MALE | 50.0 | 1201 | 5.25 |

**Table S3** The total length and number of markers in each of the linkage group for family-specific maps.

| **LG** | **Family** | **Sex** | **Length (cM)** | **Markers** | **Recombination rate (cM/Mb)** |
| --- | --- | --- | --- | --- | --- |
| 1 | F1 | Sex-averaged | 108.5 | 77550 | 3.49 |
| 2 | F1 | Sex-averaged | 106.2 | 58487 | 4.00 |
| 3 | F1 | Sex-averaged | 89.2 | 39274 | 3.96 |
| 4 | F1 | Sex-averaged | 54.6 | 39008 | 3.79 |
| 5 | F1 | Sex-averaged | 77.7 | 31541 | 5.38 |
| 6 | F1 | Sex-averaged | 50.0 | 22718 | 4.12 |
| 7 | F1 | Sex-averaged | 57.7 | 16771 | 6.02 |
| 8 | F1 | Sex-averaged | 46.9 | 23153 | 5.40 |
| 9 | F1 | Sex-averaged | 41.5 | 23517 | 4.21 |
| 10 | F1 | Sex-averaged | 60.1 | 23440 | 7.44 |
| 11 | F1 | Sex-averaged | 76.3 | 10924 | 6.92 |
| 12 | F1 | Sex-averaged | 44.6 | 7060 | 4.21 |
| 13 | F1 | Sex-averaged | 75.8 | 4293 | 9.76 |
| 14 | F1 | Sex-averaged | 71.7 | 2673 | 10.13 |
| 15 | F1 | Sex-averaged | 45.4 | 1560 | 4.77 |
| 16 | F1 | Sex-averaged | 62.2 | 28 | 6.44 |
| 1 | F2 | Sex-averaged | 104.3 | 84181 | 3.35 |
| 2 | F2 | Sex-averaged | 97.0 | 65011 | 3.65 |
| 3 | F2 | Sex-averaged | 75.6 | 45634 | 3.36 |
| 4 | F2 | Sex-averaged | 50.6 | 45049 | 3.51 |
| 5 | F2 | Sex-averaged | 70.1 | 35644 | 4.85 |
| 6 | F2 | Sex-averaged | 52.4 | 32016 | 4.32 |
| 7 | F2 | Sex-averaged | 47.6 | 28548 | 4.96 |
| 8 | F2 | Sex-averaged | 57.9 | 26057 | 6.66 |
| 9 | F2 | Sex-averaged | 48.2 | 24906 | 4.88 |
| 10 | F2 | Sex-averaged | 48.8 | 24114 | 6.04 |
| 11 | F2 | Sex-averaged | 86.8 | 14544 | 7.88 |
| 12 | F2 | Sex-averaged | 56.7 | 9867 | 5.35 |
| 13 | F2 | Sex-averaged | 63.4 | 4852 | 8.16 |
| 14 | F2 | Sex-averaged | 51.8 | 2886 | 7.32 |
| 15 | F2 | Sex-averaged | 52.5 | 1722 | 5.51 |
| 16 | F2 | Sex-averaged | 64.8 | 128 | 6.72 |

**Table S4.** Percentage of repeats per linkage group (LG).

| **LG** | **Repeats (%)** |  |
| --- | --- | --- |
| LG1 | 45.91 |  |
| LG2 | 52.16 |  |
| LG3 | 56.94 |  |
| LG4 | 25.25 |  |
| LG5 | 59.48 |  |
| LG6 | 23.53 |  |
| LG7 | 47.08 |  |
| LG8 | 36.18 |  |
| LG9 | 43.85 |  |
| LG10 | 56.97 |  |
| LG11 | 48.26 |  |
| LG12 | 71.52 |  |
| LG13 | 68.21 |  |
| LG14 | 65.53 |  |
| LG15 | 79.75 |  |
| LG16 | 24.14 |  |

**Table S5** List of the Eurasian spruce bark beetle contigs altered during the assembly process (e.g., trimmed, split, or rearranged).

| **Contig affected** | **What happened?** | **LG affected** | **Inversion status** | **Additional info** |
| --- | --- | --- | --- | --- |
| IpsContig6 | part of the contig was removed and did not assign to any other LG | LG5 | yes | 29434 bp removed |
| IpsContig6 | contig rearranged within LG | LG5 | no | cut and rearranged |
| IpsContig5 | contig rearranged within LG | LG6 | yes | cut and rearranged |
| IpsContig65 | contig cut into fragments that ended up in different LGs | LG5, LG12 | no |  |
| IpsContig14 | contig rearranged within LG | LG10 | yes | only gaps introduced |
| IpsContig41 | contig cut into fragments that ended up in different LGs | LG10, LG11 | no |  |
| IpsContig16 | contig rearranged within LG | LG11 | yes | cut and rearranged |
| IpsContig23 | contig rearranged within LG | LG11 | yes | cut and rearranged |
| IpsContig29 | contig cut into fragments that ended up in different LGs | LG12, LG14 | yes |  |
| IpsContig18 | part of the contig was removed and did not assign to any other LGs | LG13 | yes | 160 bp removed |
| IpsContig18 | part of the contig was removed and did not assign to any other LGs | LG13 | yes | 62 bp removed |
| IpsContig18 | contig rearranged within LG | LG13 | yes | cut and rearranged |
| IpsContig51 | contig rearranged within LG | LG13 | yes | cut and rearranged |
| IpsContig26 | contig rearranged within LG | LG14 | yes | cut and rearranged |
| IpsContig31 | part of the contig was removed and did not assign to any other LGs | LG16 | no | 196690 bp removed manually from LG16 together with IpsContig33 that is likely associated with mtDNA, most of this contig is part of LG12 |
| IpsContig35 | part of the contig was removed and did not assign to any other LGs | LG16 | no | 498302 bp removed |
|  |  |  |  |  |

**Table S6.** Information on how inversions were affected by linkage map anchoring and the assembly process.

| **LG** | **Inversion ID** | **Start** | **End** | **Old size** | **New size** | **Difference in size** | **Additional information** |
| --- | --- | --- | --- | --- | --- | --- | --- |
| LG1 | Inv2 | 12700001 | 18860001 | 4.04 | 6.16 | 2.12 | IpsContig2, IpsContig259 and IpsContig3 connected |
| LG5 | Inv6 | 4460001 | 6140001 | 0.21 | 1.68 | 1.47 | small rearrangement of IpsContig6 and connection to IpsContig213, IpsContig251, and IpsContig65 |
| LG7 | Inv13 | 580001 | 5700001 | 4.5 | 5.12 | 0.62 | IpsContig13 and IpsContig43 connected |
| LG11 | Inv16.1  Inv23.1 | 3700001 | 11013495 | 6.67 | 7.28 | 0.64 | rearrangements of IpsContig16 and IpsContig23 and connection to IpsContig72 |
| LG13 | Inv18 | 1 | 3100001 | 2.32 | 3.1 | 0.78 | rearrangement of IpsContig18 and connection to IpsContig51 |
| LG14 | Inv26 | 240001 | 2300001 | 0.1 | 2.06 | 1.96 | rearrangement of IpsContig26 and connection to IpsContig 57 and IpsContig97 |
| LG10 | Inv14.2 | 5280001 | 7940001 | 0.67 | 2.66 | 1.99 | boundaries were redefined based on Fst estimates between homozygotes |
| LG16 | Inv9 | 2280001 | 5980001 | 3.3 | 3.7 | 0.4 | boundaries were redefined based on Fst estimates between homozygotes |

**Table S7.** A list of newly identified LD clusters within the linkage group, each examined for inversion-like patterns using PCA (i.e., the presence of three distinct clusters corresponding to the three possible genotypes). Refer to Figure S15 for the associated PCA plots. The start and end coordinates indicate the genomic regions that were tested. For regions showing inversion-like patterns, putative inversion boundaries were inferred based on elevated Fst values between individuals homozygous for alternate haplotypes.

| **LG** | **Start (Mb)** | **End (Mb)** | **Inversion PCA pattern** |
| --- | --- | --- | --- |
| LG2 | 8 | 11 | no |
| LG3 | 11 | 13 | no |
| LG4 | 0.5 | 1 | no |
| LG4 | 13 | 14.4 | no |
| LG7 | 0 | 0.5 | yes |
| LG8 | 0 | 0.3 | no |
| LG12 | 6 | 8 | no |
| LG15 | 8 | 8.5 | no |
| LG15 | 8.6 | 8.9 | yes |
| LG15 | 9 | 9.2 | yes |

**Figure S1.** First metphase of meiosis of male *Ips typographus*. Chromosomes form 16 bivalents, X and y_p_ indicate a sex chromosome pair. 2n = 32 (30+Xy_p_).


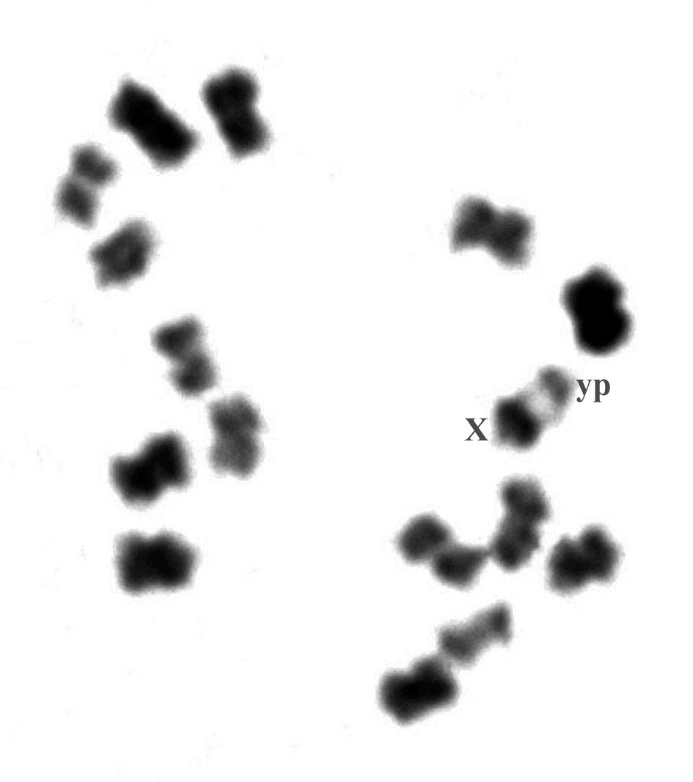


**Figure S2** The Eurasian spruce bark beetle Marey map for both families (family avaraged). Sex averaged genetic distance (cM) on the y axis against the physical distance (Mb) on the x axis for sixteen linkage groups (LGs) likely corresponding to the bark beetle chromosomes. LG1-LG15- autosomes; LG16 - X chromosome. Light grey rectangles indicate the position of polymorphic inversions based on the Mykhailenko et al. (2024) and dark grey rectangles indicate the inversion coordinates as updated in the current study.

**Figure S3.** The Eurasian spruce bark beetle Marey map for both families. Sex averaged genetic distance (y-axis, cM) along the physical distance (x-axis, Mb) in sixteen linkage groups (LGs) likely corresponding to species chromosomes. LG1-LG15- autosomes; LG16 - X chromosome. Light grey rectangles indicate the position of polymorphic inversions based on the Mykhailenko et al. (2024) and dark grey rectangles indicate the inversion coordinates as updated in the current study. Dots of different colors indicate different contigs each LGs consist of (reported in agp files in Supplementary Information). One can also see within contigs rearrangements (e.g. LD13) since contigs keep the same color even if they were cut and combined with other contigs.

**Figure S4.** Sex-specific Marey maps: LG1-LG15- autosomes; LG16 - X chromosome. Light grey rectangles indicate the position of polymorphic inversions based on the Mykhailenko et al. (2024) and dark grey rectangles indicate the inversion coordinates as updated in the current study. Dots of different colors indicate different contigs each LGs consist of (reported in agp files in Supplementary Information). One can also see within-contigs rearrangements (e.g. LD13) since contigs keep the same color even if they were cut and combined with other contigs.

**Figure S5.** Sex-specific Marey maps: male - green, female - purple. LG1-LG15- autosomes; LG16 - X chromosome. Light grey rectangles indicate the position of polymorphic inversions based on the Mykhailenko et al. (2024) and dark grey rectangles indicate the inversion coordinates as updated in the current study.

**Figure S6.** Family-specific sex-averaged Marey maps: family F1 - blue, family F2 - orange. LG1-LG15- autosomes; LG16 - X chromosome. Only female maps are shown for the LG16. Light grey rectangles indicate the position of polymorphic inversions based on the Mykhailenko et al. 2024 and dark grey rectangles indicate updated inversion coordinates.

**Figure S7** The parent-specific recombination rate (cM/Mb) along linkage groups estimated using a sliding window approach with MareyMapOnline. LG1-LG15- autosomes; LG16 - X chromosome. Light grey rectangles indicate the position of polymorphic inversions based on the Mykhailenko et al. (2024) and dark grey rectangles indicate new updated inversion coordinates. Tables include inversion physical coordinates (Mb) and associated inversion genotypes (AA, BB – homozygotes, AB- heterozygotes) to guide expectations of recombination suppression (in heterozygotes, AB).

**Figure S8.** Patterns of linkage disequilibrium, PCA, and Fst in inversion regions. POP: populations. Bluish colors indicate northern populations, yellowish colors indicate Polish populations, and brownish and reddish colors indicate southern populations. The panels are sorted by linkage group (from 1 to 16). Each inversion is shown in a separate panel, which displays the linkage disequilibrium pattern in the linkage group, the PCA for the inversion region, and the Fst between the alternative inversion homozygotes. Red bars indicate the location of the inversion. Additional PCA plots are shown in some cases, e.g., when PCA inversion-like patterns are only visible in northern (or southern) populations. Green bars below the Fst plots indicate the position of nanopore inferred inversions (only inversions >10kb are plotted).


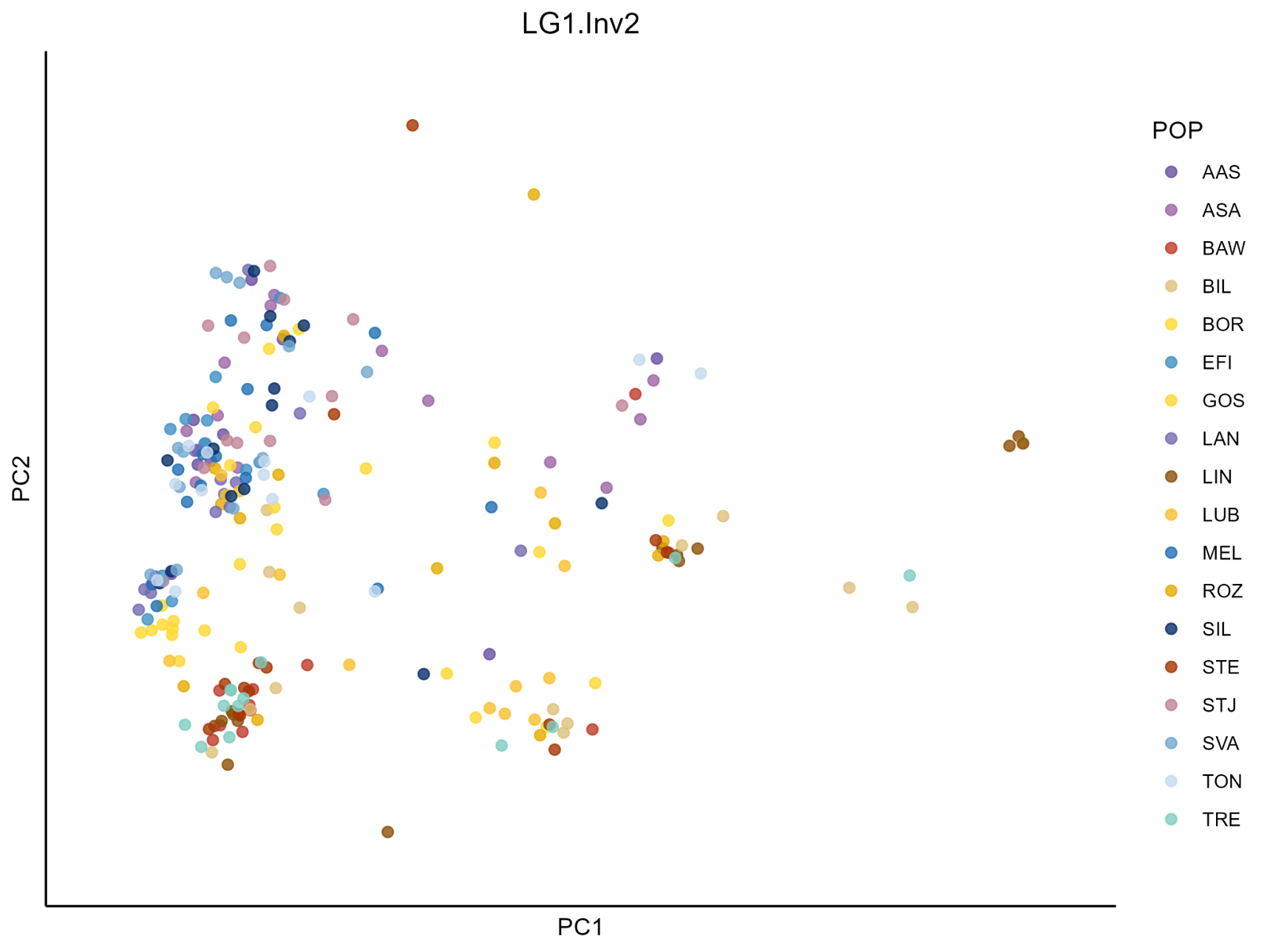


**LG1- Inv2**

PCA inversion like patterns are only visible in southern populations and in a much smaller region then indicated by linkage disequilibrium and Fst patterns (shown from 17-18 and 18 -19 Mb).

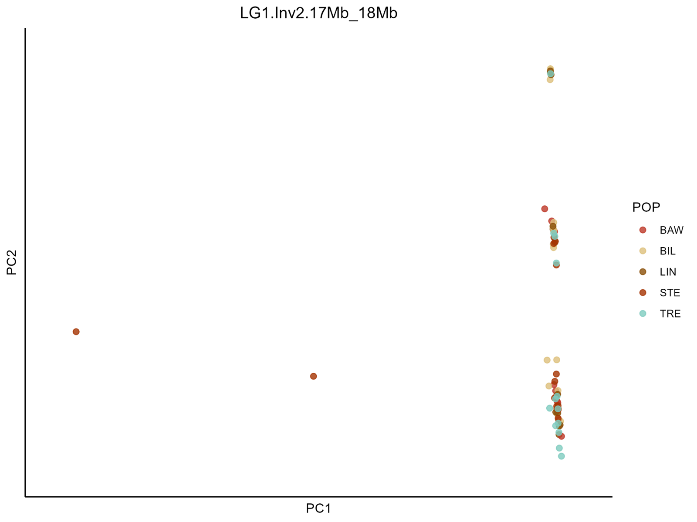

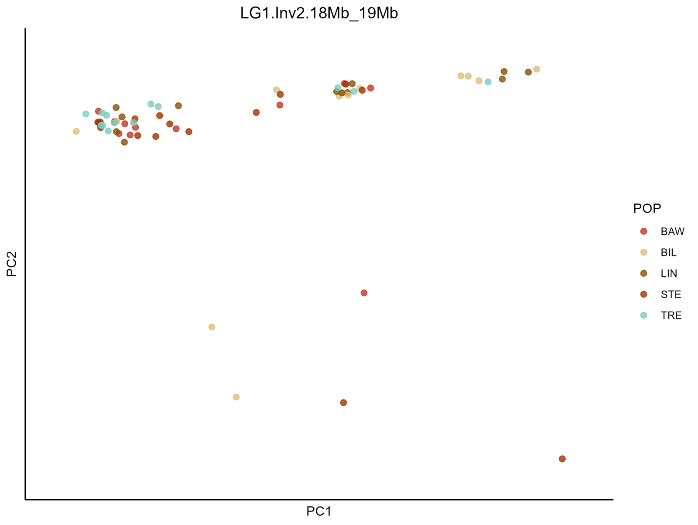


**LG1- Inv3**

**LG2 – Inv12**

**LG3 – Inv15**

**LG4 – Inv10**

**LG5 -Inv17**

**LG5- Inv6**

**LG6 - Inv5**

**LG7 – Inv13**

**LG7 - InvLG7**

**LG9 - Inv7.1**

**LG9 – Inv7.2**

**LG10 – Inv14.1**

**LG10 - Inv14.2**

**LG10 – Inv14.3**

**LG10 – Inv14.4**

**LG10 – Inv14.5**

**LG10 - Inv14.6**

**LG11 - Inv16.1+23.1**

**LG11 - Inv16.2+23.2**

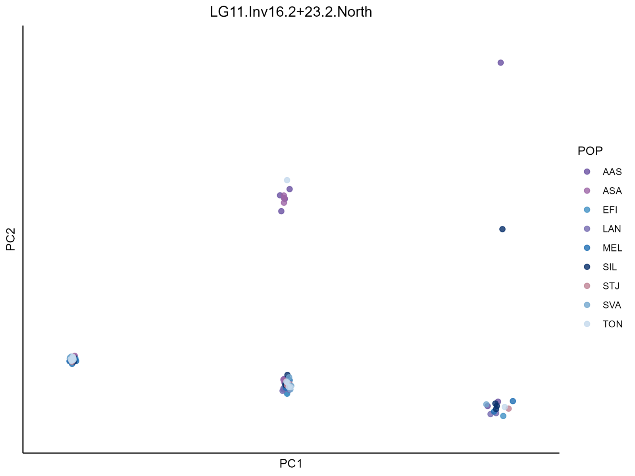


**LG12 - Inv22.1**

**LG12 - Inv22.2**

**LG12 - Inv22.3**

**LG12 -Inv22.4**

**LG12 – Inv22.5**

**LG13 - Inv18**

**LG14 – Inv26**

**LG16 – Inv9**

LG15 – **InvLG15.1**

**LG15 – InvLG15.2**

**Figure S9** The Eurasian spruce bark beetle recombination rate (cM/Mb) along linkage groups estimated using a sliding window approach with MareyMapOnline and using family-averaged map. LG1-LG15- autosomes; LG16 - X chromosome. Light grey rectangles indicate the position of polymorphic inversions based on the Mykhailenko et al. (2024) and dark grey rectangles indicate new updated inversion coordinates.

**Figure S10.** Correlation between linkage group (LG) size and mean LG recombination rate and between linkage group physical size and its map length. The correlations are done for sex and family-averaged linkage map.

**Figure S11.** Correlation between linkage group (LG) size and mean LG recombination rate and between linkage group physical size and its map length. The correlations are done for family-specific, sex-averaged linkage maps.

**Figure S12.** Correlation between linkage group (LG) size and mean LG recombination rate and between linkage group physical size and its map length. The correlations are done for parent-specific linkage maps.

**Figure S13.** Comparison of the inferred recombination events in heterozygous versus homozygous parents. To test whether heterozygous individuals exhibit reduced recombination, recombination event counts were weighted by the number of offspring produced by each parent prior to the test. This means that for each inversion and each parent the observed number of recombination events were divided by either 65 (family F1) or 82 (family F2).

**Figure S14.** Repeat content in the Eurasian spruce bark beetle genome

**Figure S15.** PCA results for the new linkage disequilibrium clusters discovered in the linkage disequilibrium analysis. See Table S4 for a list of investigated regions.

**LG2: 8Mb – 11Mb**

**LG3: 11Mb – 13Mb**

**LG4: 0.5Mb – 1Mb**

**LG4: 0.5Mb – 1Mb – Northern populations**

**LG4: 13Mb – 14.4Mb**

**LG7: 0Mb – 0.5Mb**

**LG8: 0Mb – 0.3Mb**

**LG12: 6Mb – 8Mb**

**LG15: 8Mb – 8.5Mb**

**LG15: 8.6 – 8.9Mb**

**LG15: 9Mb – 9.2Mb**

**Figure S16.** Comparison of recombination rate between collinear (green) and inversion regions (yellow) per linkage group. This plot is based on the results from MareyMapOnline.

**Figure S17.** Density of all repeats along the 16 LGs. Density was quantified in 50 kb sliding windows by calculating the total length of repeat sequences within each window divided by the window size. Grey bars indicate the positions of chromosomal inversions.

**Figure S18.** Gene density per 50 kb windows along LGs. Grey bars indicate the positions of chromosomal inversions.
